# Dahuang Zhechong Pills Suppress Silicosis Fibrosis Progression via p38 MAPK/TGF-*β*1/Smad Pathway *In Vitro*

**DOI:** 10.1155/2021/6662261

**Published:** 2021-04-02

**Authors:** Li-Juan Wu, Xiao-Yan He, Wen-Xiang Wang, Jie Liang, Yu-Die Zhang, Jing-Tao Liang, Da-Yi Chen

**Affiliations:** ^1^College of Public Health, Chengdu University of Traditional Chinese Medicine, Chengdu 610075, China; ^2^College of Basic Medicine, Chengdu University of Traditional Chinese Medicine, Chengdu 610075, China; ^3^College of Pharmacy, Chengdu University of Traditional Chinese Medicine, Chengdu 610075, China; ^4^College of Clinical Medicine, Chengdu University of Traditional Chinese Medicine, Chengdu 610036, China

## Abstract

**Background:**

Dahuang Zhechong pills (DHZCP) is a classic Chinese medicinal prescription in “Treatise on Cold Pathogenic and Miscellaneous Diseases (Shanghan Zabing Lun),” and it has the function of tonifying blood, nourishing Yin, and removing blood stasis. Previous studies have shown that DHZCP could alleviate SiO_2_ induced pulmonary fibrosis in mice. This study aims to further explore the preventive and therapeutic effects of DHZCP against silicosis fibrosis and the underlying mechanisms *in vitro*.

**Methods:**

We used the experimental model of SiO_2_-induced MH-S cells to evaluate the therapeutic potential of DHZCP. MH-S cells induced by SiO_2_ were intervened with the drug-containing serum of DHZCP, and the effects of drug-containing serum of DHZCP on the MH-S cells were detected by CCK8, ELISA, flow cytometry, western blot, and immunofluorescence.

**Results:**

DHZCP improved cell viability by reducing apoptosis. It also decreased the levels of TNF-*α*, IL-1*β*, IL-6 in the supernatant of MH-S cells induced by SiO_2_, inhibited the expression of p38 MAPK, blocked the activation of NF-*κ*B, and controlled the upstream inflammatory response by multiple targeting. Concomitantly, we observed upregulation of Smad7 and a marked decline in TGF-*β*1, *α*-SMA, Smad2, Smad3 expression in MH-S cells treated with DHZCP.

**Conclusion:**

To sum up, we conclude that DHZCP protects against SiO_2_-induced silicosis by reducing the persistent irritation of inflammation, regulating the p38 MAPK/TGF-*β*1/Smad pathway.

## 1. Introduction

Silicosis, one of the most common occupational diseases in the world is an irreversible and progressive interstitial pulmonary disease caused by long-term inhalation and retention of free silica (SiO_2_) [1–3]. Despite the continuous exploration and progress in the prevention of silicosis, there were still many new cases every year [4]. This disease has brought a huge social and economic burden to the world, especially to developing countries [5].

The pathological processes of silicosis start from the phagocytosis of SiO_2_ by macrophages, which leads to an inflammatory cascade, excessive proliferation and migration of fibroblasts, and gradual development of fibrosis [6]. Its pathological features include early infiltration of inflammatory cells, persistent pulmonary inflammation, excessive deposition of extracellular matrix (ECM), interstitial fibrosis, and formation of silicon nodules [7]. Though the pathogenesis and prevention of silicosis have been explored for many years, the exact and crucial pathogenesis has not been fully elucidated [8, 9]. In terms of treatment, there are no specific targets for screening and diagnosis of silicosis in the early stage. Once silicosis is diagnosed, it is in the late stage of irreversible fibrosis, and effective drugs for its treatment are still lacking [6]. Therefore, it is necessary to find the potential prevention and therapeutic methods and drugs of silicosis intervention [9].

Dahuang Zhechong pills (DHZCP), a canonical traditional Chinese medicine from “Treatise on Cold Pathogenic and Miscellaneous Diseases (Shanghan Zabing Lun),” and consisting of *大黄* (*Rheum palmatum L.*), *黄芩* (*Scutellaria baicalensis Georgi*), *甘草* (*Glycyrrhiza uralensis Fisch*), *桃仁*(*Persicae Semen*), *杏仁* (*Amygdalus Communis Vas*), *芍藥*(*Paeonia lactiflora Pall.*),*幹地黃* (*Rehmannia glutinosa* (*Gaertn.*) *Li-bosch*.), *幹漆*(*Toxicodendri Resina*), *虻蟲* (*Tabanus*), *水蛭* (*Hirudo*), *蠐螬* (*Holotrichiae larva*), and *黁蟲*(*Eupolyphaga*), are officially recorded in Chinese Pharmacopoeia [10] and is clinically used to treat gynecopathy, hepatic diseases, and atherosclerosis [11]. However, researches on the treatment of silicosis with DHZCP have never been reported except in our previous study, wherein we preliminary evidenced that DHZCP was effective on mice with silicosis induced by SiO_2_ [12]. Therefore, we conducted extensive experiments *in vitro* to further explore the preventive and therapeutic effects of DHZCP on silicosis and the mechanisms.

## 2. Materials and Methods

### 2.1. Preparation of Drugs and SiO_2_ Suspension

DHZCP (17013174) was purchased from Beijing Tongrentang Co., Ltd. (Tongrentang pharmaceutical factory, Beijing, China), and the executive standard is the Pharmacopoeia of the People's Republic of China (Volume 1, 2015 Edition). Twelve hours before intragastric administration, DHZCP was ground with normal saline to make suspension with a concentration of 2 g/mL, which was stored at 4°C.

Pyrrolidine dithiocarbamate ammonium (PDTC, CAS: 5108-96-3) is an NF-*κ*B inhibitor. It was purchased from MedChemExpress (Monmouth Junction, NJ, USA). Its purity is 99.86% and the specification is 10 mM*∗*1 mL in DMSO. 75 *μ*L DMSO was added into 50 *μ*L PDTC (10 mM), and after diluting and mixing, a stock solution with a concentration of 4 mM was obtained and stored at −20°C for standby.

SiO_2_ was purchased from RHAWN (CAS: 60676-86-0, Shanghai, China), and the purity of the product was above 99%. The SiO_2_ dust was dried at 180°C for 2 h, and a suspension of SiO_2_ with a concentration of 20 mg/mL was prepared by mixing SiO_2_ dust with sterile phosphate-buffered saline, which was then sterilized at 121°C for 20 min and then stored at −20°C. The suspension was then diluted to 40 *μ*g/mL before use.

### 2.2. Preparation of DHZCP-Medicated Serum (DMS)

A total of 24 Sprague-Dawley male rats (SPF class; aged 5 weeks, 145–160 g) were purchased from the Chengdu Dashuo Laboratory Animal Co., Ltd. (Sichuan, China). All rats were raised in an air-conditioned room under stable temperature (23 ± 2°C) and humidity (50%–70%) with a regular 12 h light/dark cycle. The animals had unlimited access to tap water and standard food. The animal experiment in this study has been approved by the experimental animal ethics committee of Chengdu University of traditional Chinese medicine (Sichuan, China), and is in line with the guidelines for experimental animal care and use issued by the National Institute of Health.

The rats were divided into 2 groups randomly: DHZCP and control group, with 12 rats in each group. The dose for the DHZCP group was 1.35 g/kg, and the control group was given an equal volume of distilled water. Rats were fed DHZCP by gavage twice a day for 7 days. After the last gastric perfusion for 1 h, all rats were anesthetized by sodium pentobarbital. Blood was collected from the abdominal aorta of rats in a sterile environment, stood 10 min (room temperature), and then centrifuged for 15 min at 2000 r/min. The supernatant was separated and inactivated at 56°C for 30 min, filtered with 0.22 *μ*m microporous membrane, the DHZCP-medicated serum (DMS) was obtained. No adverse events occurred during the experiment.

### 2.3. Determination of 5 Components in DMS Based on Q-Orbitrap High Resolution Liquid/Mass Combination

#### 2.3.1. Preparation of Test Solution

Add 1 mL methanol to 200 *μ*L DMS, mix well, and centrifugate at 4°C, 20000*g* for 10 min, take out the supernatant for detection.

#### 2.3.2. Preparation of Reference Standard Solution

Accurately weigh amygdalin, baicalin, catalpol, emodin, and paeoniflorin standard products, add appropriate amounts of pure methanol to them, and prepare a stock solution with a final concentration of 2 mg/mL. Then use pure methanol stock solution to prepare these 5 analytes into a 500 ng/mL mixed standard, and start the sample injection analysis.

#### 2.3.3. Mass Spectrometry Conditions Preparation

Ion source: electrospray ionization source (ESI); scanning method: positive and negative ion switching scanning; detection method: parallel reaction monitoring (1 A targeted protein quantification method based on Orbitrap mass spectrometry); Spary Voltage: 3.8 kV (Positive); Capillary Temperature: 300°C; Collision gas: high purity argon (purity ≥99.999%); Sheath gas: nitrogen (purity ≥99.999%), 40 Arb; auxiliary gas (Aux gus heater temp): nitrogen (purity ≥99.999%) 350°C; data collection time: 10.0 min. The conditions are shown in Table 1.

#### 2.3.4. Chromatographic Conditions

Column: RP-C18 150 × 2.1 mm 1.8 *μ*m, Welch. Flow rate: 0.30 mL/min. Aqueous phase: 0.1% formic acid aqueous solution. Organic phase: 0.1% formic acid acetonitrile. Column oven temperature: 35°C. Automatic injector temperature: 10.0°C. Autosampler needle washing volume: 200.00 *μ*L. Immersion time during needle washing of automatic injector: 3.00 ms. Autosampler injection volume: 5.00 *μ*L. The chromatographic conditions of mass spectrometry are listed in Table 2.

The test was repeated 3 times. Data chromatogram acquisition and integration of high-resolution liquid/Mass Combination were processed by Xcalibur 4.1 (Thermo), and linear regression was performed with an equal weighting coefficient.

### 2.4. Cell Line and Cell Culture

The mouse alveolar macrophage cells (MH-S) used in our study were purchased from Beina Chuanglian Biology Research Institute (Henan, China). MH-S cells were cultured in 10% fetal bovine serum (FBS, 10099141C, Gibco, Waltham, MA) and RPMI-1640 culture medium (C11875500BT, Gibco, Waltham, MA) and 1% 100 U/100 mL penicillin/streptomycin (SV30010, HyClone, Utah, USA). The culture conditions were as follows: 5% CO_2_, 37°C.

### 2.5. Cell Grouping, Poisoning, and Treatment

The cells were divided into 5 groups: model control group [13] (MC, SiO_2_ suspension, 40 *μ*g/mL), DMS + PDTC group (40 *μ*g/mL SiO_2_ suspension +15% DMS + 4 mM PDTC), DMS group (40 *μ*g/mL SiO_2_ suspension +15% DMS), PDTC group (40 *μ*g/mL SiO_2_ suspension +4 mM PDTC), and normal control group (NC, 15% drug-free serum).

The cells were inoculated into 6-well plates with 5 × 10^5^ cells per well, and then the culture medium containing FBS was replaced by the medium without serum. 4 mM PDTC was added into DMS + PDTC group and PDTC group, and the mixture was rested for 12 h. And then, SiO_2_ suspension was added to the cells in the MC, DMS + PDTC, DMS, and PDTC with a final concentration of 40 *μ*g/mL. Each plate was shaken gently and evenly and incubated for 4 h, 15% DMS was added to DMS + PDTC group and DMS group, respectively, after continuous cultivation for 12 h, the cells were taken out for subsequent experiments.

### 2.6. Cell Viability Was Measured

4 mM PDTC was added into DMS + PDTC group and PDTC group and the mixture was rested for 12 h. In addition to the NC, the other wells were given 40 *μ*g/mL SiO_2_ suspension. The cells were taken out after 4 h in the incubator. 15% DMS was added to DMS + PDTC group and DMS group and the mixture was rested for 12 h. Thereafter, 10 *μ*L of CCK8 test reagent (C0037, Shanghai, China) was added to each well, and then the culture was continued for 2 h. Using an ELISA microplate reader (BioTeK Epoch, USA) to measure the optical density (OD) value at 450 nm.

### 2.7. Flow Cytometry Analysis

The cell suspension in each well was replaced with 200 *μ*L fresh 1× binding buffer. Then, annexin V-FITC staining fluorescent dye (BMS500fi-300, Waltham, MA) was added 5 *μ*L per well for 10 min; 10 *μ*L PI staining was added, 5 min later; 400 *μ*L binding buffer was added, mixed fully and detected immediately. The data were analyzed using Cytoflex flow cytometry (Backman, USA) and Kaluza 2.1 software.

### 2.8. Determination of Cytokines

The ELISA kits of TNF-*α* (1102589), IL-1*β* (1102590), and IL-6 (1099199) were obtained from Shanghai Enzyme-Linked Biotechnology Co., Ltd. (Shanghai, China). The enzyme plate was sealed after adding the sample and incubated at 37°C for 30 min, washed 5 times with detergent, and incubated at 37°C for 30 min with the HRP labeled enzyme labeled reagent. The plate was washed 5 times again, and the color reagent was applied 20 min later and the reaction was stopped by adding the termination solution. OD at 450 nm was analyzed.

### 2.9. Western Blot Analysis

Use the BCA protein assay kit (P0009, Shanghai, China) to detect protein concentration. First, extract the protein: take out the sample to be tested, add RIPA lysis solution to each EP tube containing the tissue to be tested, and place it on crushed ice for 10 minutes; collect the lysis solution and centrifuge for 10 minutes at 4°C and 12000 rpm; take the clear liquid to be tested. Second, protein content determination: use BCA protein quantification kit to determine protein concentration. Dilute 2 *μ*L of the sample to be tested to 16 *μ*L with lysate, then add 2 *μ*L of the diluted protein to the well, add lysate to make up to 20 *μ*L, add BCA working solution, mix well, and incubate at 37°C for 30 minutes. With No. 0 hole as the control, measure the OD value of the sample at the wavelength of 562 nm; draw a standard curve based on the value obtained from the standard and calculate the protein content of the corresponding sample. After electrophoresis, the proteins were separated by 10% SDS-PAGE and transferred to a polyvinylidene fluoride membrane. Being sealed in 5% skimmed milk for 1 h at room temperature, the diluted primary antibody was incubated at 4°C overnight. The primary antibodies including p38 mitogen-activated protein kinase (p38 MAPK, 1 : 200, SC-7973, Santa Cruz, CA), nuclear factor kappa B (NF-*κ*B) p65 (1 : 200, SC-514451, Santa Cruz, CA), transforming growth factor-beta1 (TGF-*β*1, 1 : 1000, GB11179, servicebio, Wuhan), alpha-smooth muscle actin (*α*-SMA, 1 : 1000, GB13044, servicebio, Wuhan), Smad2 (1 : 1000, GB11511, Servicebio, Wuhan), Smad3 (1 : 1000, bsm-52224r, bioss, Beijing), and Smad7 (1 : 200, SC-365846, Santa Cruz, CA). After being washed 3 times with Tris-Buffered Saline Tween-20, the membranes were incubated with secondary antibody (1 : 5000, ab6721, Abcam, Cambridge) at room temperature for 2 h, then washed 3 times again. After mixing A and B reagents of enhanced chemiluminescence luminescent solution (ECL, KF001, Affinity Biosciences, OH), drop them onto the membrane uniformly for 1 minute and then put the membrane into the dark room of the chemiluminescence gel imager. Images were captured with a Gene Gnome Gel Imaging System (Syngene, USA) and Image J Software was used to analyze the gel images.

### 2.10. Immunofluorescence Staining

The antibodies are as described above. After the slides of cells were spin-dried, 50–100 *μ*L of membrane breaking working solution was added and incubated 20 min, then it was washed with phosphate-buffered saline for 3 times. The cells were covered with 3% bovine serum albumin, sealed 30 min, and then incubated overnight with primary antibody at 4°C. The next day, sections were incubated with fluorescein isothiocyanate-conjugated secondary antibody for 50 min at room temperature, after dripping of 4′,6-Diamidino-2-phenylindole (DAPI, G1012, OH, USA) dye solution and were incubated in the dark for 10 min. Images were photographed by fluorescent microscopy (Nikon Eclipse C1, Japan).

### 2.11. Statistical Analysis

The data in this experiment are analyzed using SPSS25.0 software. The data are presented as the mean ± SEM. Intragroup comparison: paired *T*-test is used for normal distribution, and rank sum test is used for nonconforming distribution. Comparison between groups: one-way analysis of variance (One-Way ANOVA method) is used for normal distribution, LSD test is used for uniform variance, Tamhane's T2 test is used for uneven variance, rank sum test is used for nonnormal distribution. *P* < 0.05 is considered to be a statistically significant difference, *P* < 0.01 is considered a significant difference.

## 3. Results

### 3.1. The Contents of 5 Components of Traditional Chinese Medicine in DMS

**Figure 1:**
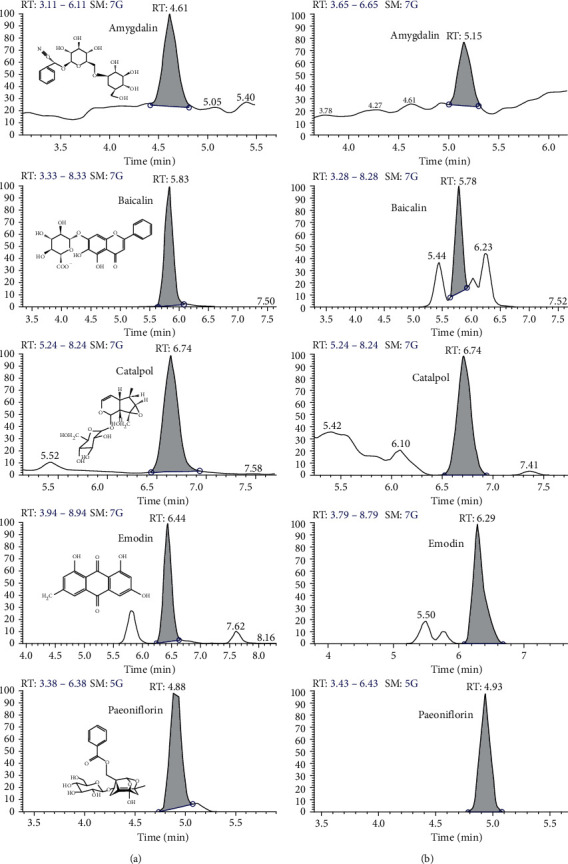
Representative chromatograms of reference standard solution and DMS sample. (a) Representative chromatograms of the five compounds in reference standard solution. From top to bottom: amygdalin (500 ng/mL), baicalin (500 ng/mL), catalpol (500 ng/mL), emodin (500 ng/mL), paeoniflorin (500 ng/mL). (b) Representative chromatograms of the five compounds in the DMS sample. From top to bottom: amygdalin, baicalin, catalpol, emodin, paeoniflorin.

The results of the reference standard compounds and DMS samples were shown in Figure 1, and the contents were shown in Table 3. 5 major components of DMS were verified by comparing individual peak retention time and quantifier ions with the reference standard compounds. The peak area of amygdalin, baicalin, catalpol, emodin, and paeoniflorin were taken as the vertical coordinate and the concentration as the horizontal coordinate, and the standard curve was obtained by regression with the weighted coefficient (Equal).

### 3.2. DHZCP Alleviates Impaired Cell Viability

As shown in Table 4, 40 *μ*g/mL SiO_2_ significantly decreased the MH-S cell viability compared with normal cells. However, 15% DMS and 4 mM PDTC effectively improved the viability of MH-S cells to different degrees, especially the combination of DMS and PDTC, suggesting that DHZCP reduced the cell damage caused by SiO_2_. It can be seen from the data statistics that compared with the NC group, the cell viability of MC was significantly impaired, and there was a significant difference (*P* < 0.001). Compared with MC in DMC + PDTC and DMS groups, cell viability was significantly restored (*P* < 0.001 and *P* < 0.01). Compared with the MC group, the PDTC group showed a tendency to restore cell viability.

### 3.3. DHZCP Inhibits SiO_2_-Induced Apoptosis in MH-S Cells

**Figure 2:**
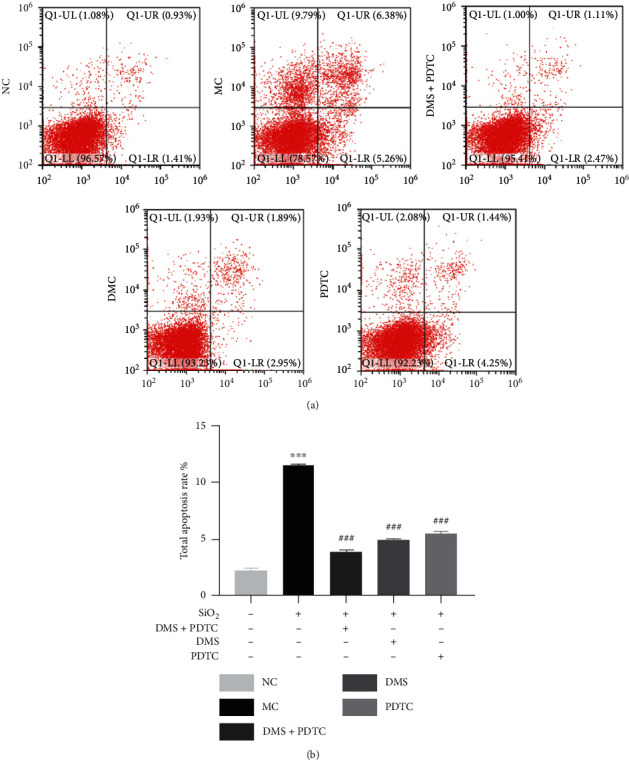
DHZCP inhibits apoptosis in MH-S after SiO_2_ exposure. (a) Two dimensional scatter diagram of 5 groups. (b) Repeat the experiment 3 times and count the total apoptosis rate (%). *n* = 3, ^*∗∗∗*^*P* < 0.001 VS the NC group, ^###^*P* < 0.001 vs. the MC group.

As Figure 2 shows, apoptosis significantly increased under stimulation of SiO_2_, as expected. In the MC group, the total apoptosis rate was 11.56%. Compared with the MC group, the DMS group and PDTC group significantly reduced the apoptosis rate. However, DMS and PDTC combined treatment was more effective, as the total apoptosis rate was only 3.87%. Compared with PDTC group, the total apoptosis rate of the DMS group was lower than the PDTC group, which indicated that DMS was better than PDTC in reducing apoptosis.

### 3.4. DHZCP Reduces the Level of Inflammatory Cytokines in Cell Supernatant

As illustrated in Table 5, compared with normal MH-S cells, the levels of TNF-*α*, IL-1*β,* and IL-6 in the MC group were significantly increased, and TNF-*α* and IL-1*β* were significantly increased (*P* < 0.001), and there were significant differences. After DMS and PDTC treatment, the contents of TNF-*α*, IL-1*β,* and IL-6 in the cell supernatant were significantly reduced. Among them, TNF-*α* was significantly different in the DMS + PDTC group, DMS group, PDTC group, and the model group (*P* < 0.001). Compared with the model group, the content of IL-1*β* is different in the DMS group (*P* < 0.01), and the difference is significant (*P* < 0.001) in the DMS + PDTC group and the PDTC group (*P* < 0.001), and the content of IL-6 is present compared with the model group; it showed a downward trend, but it was not statistically significant.

### 3.5. DHZCP Regulates the Proteins Expression of p38 MAPK/TGF-*β*1 Pathway

**Figure 3:**
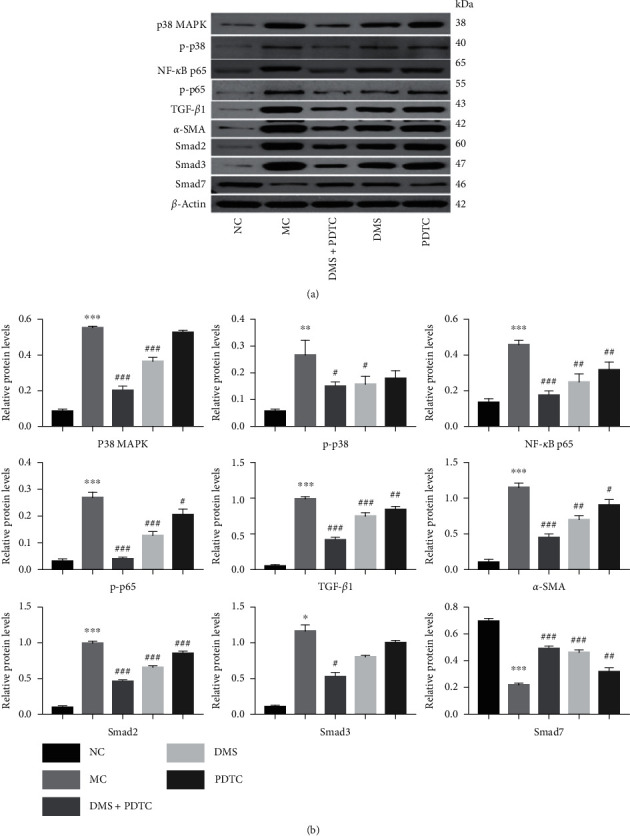
DHZCP regulates the protein expression of the p38 MAPK/TGF-*β*1/Smad pathway in MH-S cells induced by SiO_2_. (a) Representative western blot bands of p38 MAPK, p-p38, NF-*κ*B p65, p-p65, TGF-*β*1, *α*-SMA, Smad2, Smad3 and Smad7. (b) Quantitation of p38 MAPK, p-p38, NF-*κ*B p65, p-p65, TGF-*β*1, *α*-SMA, Smad2, Smad3, and Smad7 in MH-S. The experiment was repeated three times. *n* = 3. ^*∗*^*P* < 0.05 and ^*∗∗∗*^*P* < 0.001 VS the NC group, ^#^*P* < 0.05, ^##^*P* < 0.01 , and ^###^*P* < 0.001 VS the MC group.

**Figure 4:**
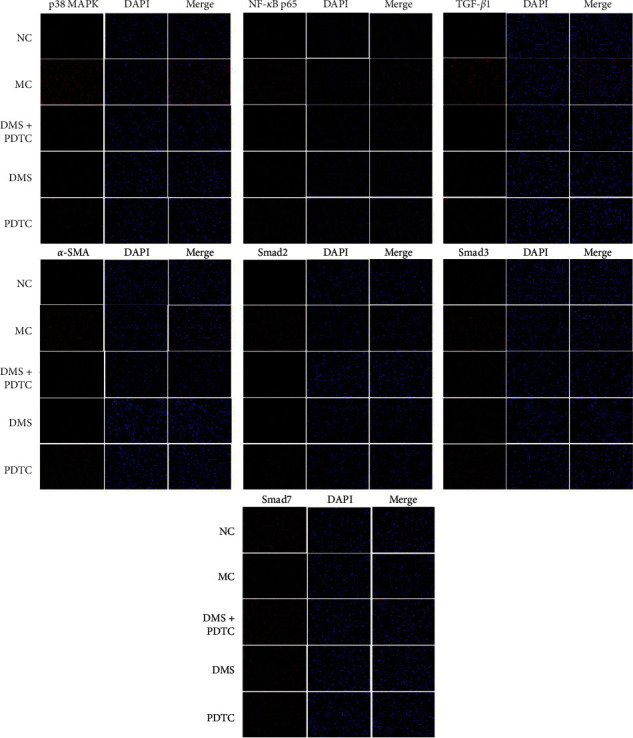
DHZCP regulates the protein average optical of p38 MAPK/TGF-*β*1/Smad pathway in MH-S cells induced by SiO2. Representative immunofluorescence staining of p38 MAPK, NF-*κ*B p65, TGF-*β*1, *α*-SMA, Smad2, Smad3, and Smad7 (red) in MH-S. Nuclei were stained with DAPI (the blue mark in the pictures); Scale bar = 20 *μ*m.

As in Figure 3, compared with normal cells, the protein expression of p38 MAPK, NF-*κ*B p65, TGF-*β*1, *α*-SMA, Smad2, and Smad3 in MH-S cells induced by SiO_2_ increased significantly. Among them, the increase of p38 MAPK, NF-*κ*B p65, p-p65, TGF-*β*1, *α*-SMA, Smad2 was significantly different from the model group (*P* < 0.001); the increase of p-p38 and Smad3 was also statistically significant (*P* < 0.01, *P* < 0.05). On the contrary, the model Smad7 of the group was significantly lower than that of the normal group (*P* < 0.001). However, after DMS treatment, the expression of these proteins significantly decreased. The trend of Smad7 protein expression was the opposite. Interestingly, PDTC, an inhibitor of NF-*κ*B, was used to interfere with SiO_2_-induced MH-S cells. In addition to the protein of p38 MAPK, p-p38, PDTC also significantly decreased the expression of NF-*κ*B p65, p-p65, TGF-*β*1, *α*-SMA, Smad2, Smad3 proteins and increased the expression of Smad7 protein. Moreover, the combined effect of PDTC and DMS was better than that of the two being used separately, except for smad3, there are significant differences between other indicators and the model group (*P* < 0.001). And this regulatory effect of DHZCP in SiO_2_-induced MH-S cells was further confirmed by immunofluorescence staining, as indicated in Figure 4.

## 4. Discussion

The aim of this study was to explore the preventive and therapeutic effects of DHZCP on silicosis and the mechanisms. Persistent inflammatory stimulation is an important cause of fibrosis [14]. The inhaled SiO_2_ particles were phagocytized by alveolar macrophages because when the body experiences an inflammatory response, it activates a natural immune response, and the activated macrophages release inflammatory cytokines, which could attract more monocyte or macrophage apoptosis. As the SiO_2_ clearance function was impaired, lung tissues gradually develop persistent pulmonary inflammation [7], alveolar macrophages, fibroblasts, and endothelial cells initiate abnormal repair patterns, leading to the deposition of the extracellular matrix, and ultimately to the formation of irreversible abnormal remodeling and fibrosis of lung tissue [15]. According to the Chinese Pharmacopoeia, we determined the contents of Amygdalin, Baicalin, Catalpol, Emodin, and Paeoniflorin in DHZCP medicated serum and then used them in the experiment to ensure the validity of the experiment. In this study, we found that DHZCP could improve vitality and significantly decrease the apoptosis rate of SiO_2_-induced MH-S cells. It was also found that DHZCP could reduce the levels of cytokines (TNF-*α*, IL-1*β*, IL-6) in cell supernatant significantly. In addition, PDTC can not only affect the DNA binding activity but also directly suppress NF-*κ*B and depress the nuclear translocation of NF-*κ*B, thereby decreasing the two subunits p50 and p65 of NF-*κ*B transferred to the nucleus to play an inhibitory role. Therefore, the expression of TNF-*α*, IL-6, and IL-1*β* genes effectively reduces the release of neutral hydrolase, peroxidase, and acid hydrolase, which are destructive to tissues from the inflammatory sites, thereby reducing the inflammatory response. Blocking NF-*κ*B activation is an effective way to control the overall pathway upstream of an inflammatory reaction and prevent organ dysfunction. p38 MAPK, the upstream gene of NF-*κ*B, could be activated by inflammation and stress reaction [16–18]. In our study, the expression of NF-*κ*B p65 and p-p65 in cells stimulated by SiO_2_ was significantly upregulated; however, they were significantly downregulated in pretreated PDTC cells stimulated by SiO_2_. In addition, the expression of p38 MAPK and p-p38 in the upstream did not change significantly, while the TGF-*β*1, Smad2, Smad3 in the downstream reduced accordingly. It shows that PDTC pretreatment could downregulate the expression of NF-*κ*B p65 and nuclear expression level of p-p65 by reducing NF-*κ*B activation so that the terminals of the pathway could not receive the stimulation signal from SiO_2_, thus inhibiting the release of the TNF-*α*, IL-1*β*, IL-6, and the development of inflammation. It also indicated that PDTC had no significant effect on the upstream p38 MAPK. When DHZCP was applied to MH-S cells activated by SiO_2_, in addition to declining the NF-*κ*B p65 and p*-*p65 to block the TGF-*β*1/Smad signaling pathway, it also significantly downregulated the expression of p38 MAPK and p-p38. It was suggested that DHZCP could inhibit inflammation and TGF-*β*1/Smad signaling pathway by blocking NF-*κ*B activation; it also could inhibit p38 MAPK upstream of NF-*κ*B. DHZCP could control the inflammatory reaction from the upstream with multiple targets, which is more effective than the single inhibition of NF-*κ*B by PDTC. This was also in line with the law of traditional Chinese medicine multitarget treatment of diseases. As mentioned above, DHZCP could decrease the levels of inflammatory cytokines in MH-S cells induced by SiO_2_. These results indicated that DHZCP could effectively reduce inflammation in the early stage of silicosis and eliminate or alleviate the continuous inflammatory stimulation of SiO_2_.

NF-*κ*B, an upstream regulatory gene for TGF-*β*1 expression, when NF-*κ*B is activated, can induce the expression of TGF-*β*1 [19]. In the case of inflammation, NF-*κ*B is phosphorylated and transferred to the nucleus to become a nuclear transcription factor, and it induces the expression of TGF-*β*1 [19]. It can be seen that in the model group, with the increase of p-p65, the expression of TGF-*β*1 also has an upward trend. On the contrary, after using DHZCP, NF-*κ*B p65, p-p65, and TGF-*β*1 showed a downward trend. TGF-*β*1 is recognized as the most important initiator of pulmonary fibrosis [20–22]. TGF-*β*1 is a major fibrogenic factor that increases the synthesis of *α*-SMA and continuously activates fibroblasts into myofibroblasts with contractile function and promotes the accumulation of ECM proteins in the lung interstitium and alveolus [23]. Smad protein is downstream signal transduction and a regulatory molecule that mediates the signal transfer from cell membrane to the nucleus [24]. At present, the protein levels of TGF-*β*1, *α*-SMA, Smad2, and Smad3 in MH-S cells stimulated by SiO_2_ were significantly increased, while Smad7 was decreased, indicating that SiO_2_ stimulation successfully activated TGF-*β*1 to initiate fibrosis and activated the downstream Smad signal. After DHZCP treatment, TGF-*β*1, *α*-SMA, Smad2, and Smad3 were significantly decreased, while Smad7 was increased. These results indicated that DHZCP downregulated TGF-*β*1 and regulated the downstream Smad signaling pathway, effectively inhibiting the initiation of fibrosis. Therefore, the protective effect of DHZCP on SiO_2_ induced MH-S cells is related to downregulating TGF-*β*1 and p38 MAPK/TGF-*β*1/Smad pathway.

## 5. Conclusions

In conclusion, we provide evidence that DHZCP could reduce the inflammatory response of MH-S cells induced by SiO_2_, eliminate the sustained inflammatory stimulation, aand effectively inhibit the initiation of fibrosis. DHZCP's strong protective effects in the SiO_2_-induced silicosis fibrosis model are related to the p38 MAPK/TGF-*β*1/Smad pathway.

## Figures and Tables

**Table 1:** Mass spectrometry conditions.

Name	Charged properties	MS1	MS2	CE
Amygdalin	−	456.15120	323.09818	50
Baicalin	+	447.09220	271.05980	50
Catalpol	−	361.07660	116.92717	50
Emodin	+	271.05966	123.00784	50
Paeoniflorin	+	498.19720	179.06996	50

**Table 2:** Chromatographic conditions of mass spectrometry.

Time (min)	The proportion of aqueous phase (%)	The proportion of organic phase (%)
0.0	98	2
0.5	98	2
6.5	2	98
9.0	2	98
9.3	98	2
10.0	98	2

**Table 3:** Contents of 5 components.

Name	Molecular formula	Contents of 5 components (ng/mL)
Amygdalin	C_20_H_27_NO_11_	1269.57 ± 176.490
Baicalin	C_21_H_18_O_11_	119.76 ± 0.049
Catalpol	C_15_H_22_O_10_	237.31 ± 17.435
Emodin	C_15_H_10_O_5_	390.78 ± 3.678
Paeoniflorin	C_23_H_28_O_11_	8.80 ± 0.292

**Table 4:** The effect of DHZCP on the viability of MH-S cells.

Group	Cell viability (%)	Mean ± SEM
NC	100	100
MC	73.31	75.681 ± 1.217^*∗∗∗*^
DMC + PDTC	91.33	92.234 ± 1.390^###^
DMS	89.13	88.518 ± 1.659^##^
PDTC	91.51	86.216 ± 2.832

**Table 5:** Effect of DHZCP on inflammatory cytokines in MH-S cells.

Group	*N*	TNF-*α* (ng/L)	IL-1*β* (ng/L)	IL-6 (pg/L)
NC	4	1.54 ± 0.819	1.50 ± 0.872	0.46 ± 0.213
MC	4	27.32 ± 2.331^*∗∗∗*^	23.34 ± 0.768^*∗∗∗*^	19.30 ± 2.464^*∗*^
DMS + PDTC	4	10.60 ± 0.976^###^	14.11 ± 1.046^###^	8.65 ± 0.336
DMS	4	11.26 ± 0.759^###^	16.22 ± 1.056^##^	9.52 ± 1.070
PDTC	4	14.72 ± 1.861^###^	15.27 ± 2.103^###^	9.09 ± 0.662

^*∗*^ *P* < 0.05 and ^*∗∗∗*^*P* < 0.001 vs. the NC group, ^##^*P* < 0.01 and ^###^*P* < 0.001 vs. the MC group.

## Data Availability

The datasets used to support the findings of this study are available from the corresponding author upon request.

## Abbreviations

*α*-SMA: Alpha-smooth muscle actin
DHZCP: Dahuang Zhechong pills
DMS: DHZCP-medicated serum
ECM: Extracellular matrix
ELISA: Enzyme linked immunosorbent assay
FBS: Fetal bovine serum
IL: Interleukin
MH-S: Mouse alveolar macrophage cells
MAPK: Mitogen-activated protein kinase
NF-*κ*B: Nuclear factor kappa B
OD: Optical density
PDTC: Pyrrolidine dithiocarbamate ammonium
TNF-*α*: Tumor necrosis factor alpha
TGF-*β*: Transforming growth factor-*β*.
